# Supplementary material for: Patterns of Intron Gain and Loss in Fungi
Source: PLoS Biol. 2004 Nov 30;2(12):e422. doi: 10.1371/journal.pbio.0020422 (PMC532390; doi:10.1371/journal.pbio.0020422)
Supplement: Table S1 — Also available at http://genes.mit.edu/NielsenEtAl/. (4.3 MB ZIP). [file pbio.0020422.st001.zip › NielsenEtAl/html/517.html]

AN7463.1.NCU03257.1.MG00537.1.FG00529.1


```
 CLUSTAL W (1.82) Multiple Sequence Alignments - Introns Inserted


Sequence 1: NCU03257.1	510 aa
Sequence 2: MG00537.1	520 aa
Sequence 3: FG00529.1	513 aa
Sequence 4: AN7463.1	544 aa
Alignment Length: 544 aa
Number Identitical Residues: 289 aa
Alignment Score (without introns) 14203


MG00537.1 	-------------MSYSWIGAPAEF0NGTN-AETGGNSA1TENLNQWYQSGDQAFILVSS
NCU03257.1	-------------MSYSWFGAPTPF~NGT--TPNGGDSA1SENLNQWYQSGDQAFILVAS
FG00529.1 	-------------MSYVVPGPPTPF~NGTN-EELGGDST1TENLNQWYQSGDQAFILVSA
AN7463.1  	MASEGMAPFTPEYASYNWTGAPSDY~SSLATNELGGNSR1VENLNKWYQSGDQAYIIVAS
          	 ::.. :. :..  **   *.*: : ..  :   **:*   ****:********:*:*::

MG00537.1 	CLVLIMIPGIAFLYSGLARRKSALSLIWVVMMSFSVVIFQWYFWGYSLAFSTSTTNGFIG
NCU03257.1	CMVLIMIPGIAFLYSGLARRKSALSLIWVCMMSSSVVMFQWYFWGYSLALSSTATNGFIG
FG00529.1 	CLVLLMVPGIAFLYSGLARRKSALSMLWVVMMSFSVIVFQWYFWGFSLAFSTTSNNPFIG
AN7463.1  	AMVMLMVPGLGFLYSGLARRKSALSMIWACMGSMCVVTFQWYFWGYSLAFSPTATNGYIG
          	.:*::*:**:.**************::*. * * .*: *******:***:*.::.* :**

MG00537.1 	DLSKFGLMNTLGKPSQGSPLIPELLYSFYQ~MQFCGVTAALVIGATAERGRVVPAMVFTF
NCU03257.1	NLRHFGLMNTLGAPSPGSNLIPELLYAFYQ0MQFVGVTAALVVGATAERGRVVPAMVFIF
FG00529.1 	DLKHFGLRHVWGAPSVGSALIPALLYSFYQ~MMFCAVTAALTIGAVAERGRVIPSMVFIF
AN7463.1  	NLRNFGLMKVLADPSPGSPLVPNLLYAFYQ0MQFCAVTAAIIMGAVAERGRLLPAMVFTF
          	:* :*** :. . ** ** *:* ***:*** * * .****: :**.*****::*:*** *

MG00537.1 	FWATIVYCPLAHWVWNANGWAFKYGVLDYAGGGPVEIGSGMSALAYSWVLGRRNEKMMLN
NCU03257.1	IWATLVYCPLCCWAWNVNGWAFKYGVMDYAGGGPVEIGSGVSALAYSWVLGRRNEKMMLN
FG00529.1 	FWATLVYCPLAYWVWNPSGWGFVNGVLDYAGGVPVEIGSGMSALAYSWVLGRRNEKMMMN
AN7463.1  	VWATIVYCPIACWVWNVNGWAFNYGVLDYAGGGPVEICSGLSALAYSMVLGRRQERMMLN
          	.***:****:. *.** .**.*  **:***** **** **:****** *****:*:**:*

MG00537.1 	FRPHNISLITLGTILLWFGWLGFNGGSAFGANLRAAMACWNTCLTAMFAAMTWTLLDFRL
NCU03257.1	FRPHNISLITIGTVFLWFGWLGFNGGSAFGANLRAAMACWNSCLTAMFAAMTWTLLDYRL
FG00529.1 	FRPHNISLITLGTIFLWFGWLGFNGGSAFGANLRAVMACWNSCLTAMFAAMTWCILDFRL
AN7463.1  	FRPHNVSLILLGTVFLWFGWLGFNGGSSFGANMRATMACWNTNLTAAFGAITWVILDWRL
          	*****:*** :**::************:****:**.*****: *** *.*:** :**:**

MG00537.1 	AKKWSLVGWCSGTISGLVAATPASGFIPPWASIVLGVVTGVCCNFGTKV1KFLLRIDDSL
NCU03257.1	AKKWSLVGWCSGTISGLVAATPASGYIPPWASIILGVVTGIVCNFGTKI~KFMIGIDDAL
FG00529.1 	ARKWSMVGWCSGTISGLVAATPASGFIDPWASIILGVVAGVACNFATKV1KFLIRIDDSL
AN7463.1  	ARKWSMVGWCSGTISGLVAATPASGFLPPWASVVLGIVTGIVCNYSTKV1KYWIRIDDSM
          	*:***:*******************:: ****::**:*:*: **:.**: *: : ***::

MG00537.1 	DVFAEHYIGGVVGLFFNGFFAADYIIGLDGVNTGLVTGGFLNQNYKQLYIQIAFIVAASA
NCU03257.1	DIFAEHALGGVVGLIFNGFFAADYIIGLDGVN-QEIPGGFLNHNYKQLYIQIAYVCATMG
FG00529.1 	DVFAEHGVGGIVGLIFNAFFARGSIIGLDGVN-TGATGGFMDGNYVIIGWQIAAIVGASS
AN7463.1  	DVFAEHGVAGIVGLIFNAFFADDAIVGLDGVNTGSEMGGWLIHNWKQLYIQIAFIVAASA
          	*:**** :.*:***:**.*** . *:******:    **::  *:  :  *** : .: .

MG00537.1 	YSFVVSAILAKIIDMIPGLKLRADEKAELLGMDDDQHGEFAYDYVE~VRRDYLAWTPAER
NCU03257.1	YSFVMSAILAKGVDMIPGLHLRASEEAELLGMDDDQHGEFAYDYVE0VRRDYLAWTPAEK
FG00529.1 	YAFVMSAIIAKIIDLIPGLKLRASEEAELLGMDDDQHGEFSYDYVE~VRRDFLAWSPQRG
AN7463.1  	YSFVVSAIVAYAINAIPGLKLRASEEAELLGMDDDQLGEFAYDYVE~VRRDYLAWTPQKH
          	*:**:***:*  :: ****:***.*:********** ***:***** ****:***:* . 

MG00537.1 	DPKEDGHAV--VPQLGIEGHQTIHDN--IINGVGSVRHTPAVPNDAHAQHDSEKPKSVDR
NCU03257.1	EQRQDGDII--VPQHGIEGHQELLS---------SSRHGPTMSELPHHEEEDPREKRGST
FG00529.1 	EPAEDGDVL--EPTHGIHEHQSMMNP----SESDDARKTPSLNTPDVIAPDAVINEKHA-
AN7463.1  	DQHADGHEVPAAARYGIGEHSEMMLDGEAPTGATNSRGSEVSGVQEINVKEESQRPAPPT
          	:   **. :.: .  **  *. :   ..  .   . *             :         

MG00537.1 	SSE------
NCU03257.1	SE-------
FG00529.1 	---------
AN7463.1  	DAEKAAQGL
          	. ..::..
```
